# Effects of emergency rescue clothing on wearers' physiological and perceptual responses in hot-humid environments

**DOI:** 10.3389/fpubh.2025.1648763

**Published:** 2025-08-21

**Authors:** Yazhuo Qian, Mengqi Yuan, Ke Yan, Yayun Li, Xuefan Wang, Hao Wu

**Affiliations:** ^1^School of Mechatronics Engineering, Beijing Institute of Technology, Beijing, China; ^2^Experimental Platform for Personnel Safety Protection in Disaster Environments, Tsinghua University Hefei Institute for Public Safety Research, Hefei, China

**Keywords:** emergency rescue clothing, physiological response, thermoregulation, physiological responses, hot-humid environments

## Abstract

**Introduction:**

To improve the work efficiency and reduce heat-related illness of emergency rescue personnel, the effects of emergency rescue clothing on physiological and perceptual responses were investigated.

**Methods:**

Thirteen participants were recruited to perform human trials in a climate chamber wherein the ambient temperature and relative humidity was controlled at 35°C and 75%, and 25°C and 65%, respectively. Moreover, participants wearing emergency rescue clothing (ERC group) and T-shirts and shorts (CON group) walked at 4 and 6 km/h on a treadmill. During the trials, physiological responses and subjective responses were obtained, and then physiological strain index (PSI) and perceptual strain index (PeSI) were calculated.

**Results:**

The results showed significant differences between the ERC and the CON in parameters such as core temperature, mean skin temperature, heart rate, PSI, although some data differences were slightly. There was a positive correlation between PeSI and PSI, as well as between mean skin temperature and thermal sensation vote, with *R* values of 0.93 and 0.94 (ERC), respectively.

**Discussion:**

Correlation analysis shows that PeSI had a potential to predict PSI. This study can replace complex and cumbersome physiological indicators by calculating the perception indicators of emergency response personnel working on site, promoting the development of the safety industry engaged in certain intensity physical labor in humid and hot environments.

## Highlights

- Revealed the physiological and psychological effects of emergency rescue clothing.
- The maximum working hours when wearing emergency rescue clothing have been specified.
- Emergency rescue clothing will increase the mean skin temperature of personnel.
- The psychological changes in wearing emergency rescue suits are not so significant.

## 1 Introduction

In today's rapidly changing world, natural disasters, industrial accidents and other emergencies occur frequently, posing a serious threat to human life and property safety. From 2000 to 2019, the United Nations Office for Disaster Risk Reduction (UNISDR) documents 7,348 significant disaster events, resulting in 1.23 million fatalities, impacting over 4.2 billion individuals—many on multiple occasions—and incurring an estimated global economic loss of US$2.97 trillion (1). Emergency rescue personnel are the vanguard in dealing with these emergencies, and their safety and performance are of great significance. However, the casualties among rescue workers are one of the real issues that need to be addressed today (2). For workers in certain professions, it is crucial to envelop their bodies to protect them against external hazards, such as emergency rescue personnel and firefighters, where specific job responsibilities involve exposure to heat hazards and significant risks of biological, chemical, radiation agents, etc. (3). Personal protective equipment (PPE) has become the last line of defense to ensure the safety and health of emergency rescue personnel and firefighters (4).

The emergency rescue site is a complex and changeable environment with a variety of unexpected factors and hazardous substances. In hot-humid conditions, people easily get heat stress, causing faster heartbeats, sweating, and tiredness (5). ISO 7243 uses WBGT as an indicator for evaluating thermal stress. The standard states that when people wear clothing in summer (with a thermal resistance of 0.5 clo) and are in a resting state (metabolic rate *M* < 117 W/m^2^), the safety limit for the human body is 32–33°C (6). At the same time, when the WBGT value does not exceed 26°C and the relative humidity is controlled between 40 and 60%, the human body feels more comfortable. However, the perception of discomfort may cause rescue personnel's judgment and skills, affecting the quality and efficiency of work (7). Therefore, it is crucial to understand the various impacts of emergency rescue clothing on the human body in extreme environments. In order to ensure the safety and work efficiency of emergency rescue personnel, some scholars have conducted research related to PPE (8) investigated the performance of different materials in PPE under various stress conditions. They found that some materials had better resistance to mechanical damage but less breathable (9) studied the thermal properties of flame-retardant fabrics used in PPE and explored methods to reduce thermal stress. At present, the research on PPE not only focuses on material properties and clothing design features, but also includes the development and improvement of some special functions, such as enhancing the flame retardancy of materials (10), anti-static performance (11), and chemical resistance (12).

Improving the protection and comfort of rescue personnel clothing is a big challenge (13) discovered that the clothing structure design greatly affects the wearer's comfort, but the link between material properties and comfort was complicated and requires more research (14) studied the ergonomic design of PPE, including fit, mobility, and comfort. Other researchers have also made some attempts to improve human comfort, by studying fabric breathability, moisture permeability, and durability (15). However, most studies have not considered how high temperature and humidity together affect clothing performance, especially in emergency rescue situations where these conditions are common. Moreover, while material properties, thermal comfort, and ergonomic design have been studied, there is still a lack of understand how ERC impacts physiological responses (e.g., skin temperature and heart rate) and perceptual responses (e.g., rate of perceived exertion and thermal sensation) in extreme environments.

However, some scholars have conducted research on the physiological and psychological changes of the human body when wearing PPE. For example, (16) found that wearing PPE in hot-humid environments raises body temperature, heart rate, and lowers work efficiency and comfort. Another study by (17) showed that PPE in hot-humid conditions significantly increases heat stress (18) studied the perceptual response of firefighters wearing PPE in high-temperature environments, and evaluated parameters such as thermal management and comfort performance. Scholars have also studied the wearing of PPE by military personnel in high-temperature environments, and the results showed that it can damage cognitive abilities and increase the risk of errors (19). Although some researches have been conducted, there are still some limitations regarding the physiological and psychological effects of wearing PPE. Current research often only focuses on one aspect of the impact, such as physiological response or perception response, lacking a comprehensive consideration of both factors. Therefore, it is essential to comprehensively evaluate the physiological and perceptual responses of emergency response clothing under high temperature and high humidity conditions.

In this study, we conducted a series of controlled experiments on subjects in a climate controlled chamber, primarily to simulate the high temperatures and humidity faced by emergency responders. The study uniquely incorporated full-scale, real-condition trials, systematically examining both physiological and perceptual responses under two distinct clothing conditions: emergency rescue clothing ERC group, T-shirts and shorts CON group. Environmental temperature and humidity were rigorously regulated to replicate the demanding environments typical of emergency rescue operations. This study provides novel insights by examining the relationship between selected physiological and perceptual responses during high-heat tasks, contributing to the practical understanding of ERC design effects on human performance. Unlike most previous studies focusing solely on physiological metrics or controlled settings, our study integrates real-time perceptual indices such as PeSI to bridge laboratory data and practical perceptions. The research results provide useful ideas for the future design and evaluation of protective clothing, filling the technological gap. This includes exploring advanced breathable materials, moisture-wicking fabrics, and modular PPE systems that allow for adaptive configurations based on environmental demands. It is expected to bring new methods to alleviate heat stress, improve human centered design, and make emergency response personnel safer, more efficient, and better at working under high pressure.

## 2 Methods

### 2.1 Participants

This study recruited 13 healthy male college student volunteers to participate in the experiment, who had a certain exercise base, normal cardiorespiratory, respiratory, and mental conditions, and no physical history of' sports injuries. The selection of 13 male participants was based on a typical sample size similar to that used in thermophysiological studies, commonly used in the preliminary stage of thermal adaptation research (20). Due to the logistical and ethical challenges associated with continuous core temperature monitoring and controlled environmental exposure, small sample exploratory studies are common in this research field. The sample size selected in this article is considered sufficient to detect moderate effect sizes in the internal design of the subjects and serve as a preliminary basis for evaluating perceptual and physiological responses. Future research will include larger and more diverse samples to confirm and expand upon these findings. The recruitment start date for this study is October 10, 2023, and the end date is October 20, 2023. In order to ensure the reliability and accuracy of the data, while reducing the variability of individual responses, each participant underwent two identical experiments. Participants were prohibited from using alcohol, caffeine and nicotine for 24 h prior to the test, as well as strenuous exercise, The human experiments in this study were conducted at the Hefei Institute of Public Safety Research, Tsinghua University, and have been approved by the Scientific Research Ethics Committee of the institute. Prior to commencing the formal experimental procedures, each participant provided their written informed consent, thereby demonstrating a comprehensive understanding of the study's objectives. Detailed instructions were provided to participants regarding the completion of questionnaires designed to measure psychological parameters. These questionnaires were administered following each trial, ensuring data collection from both physiological and psychological perspectives across all experimental repetitions. Table 1 presents the physical characteristics of participants, summarized as mean ± standard deviation. The body surface area is calculated according to the Mosteller formula, which is currently the most commonly used formula and applicable to most adult individuals.

**Table 1:** Physical characteristics of the participants.

**Characteristics**	**M ±SD**	**Range**
Age (years)	21 ± 1	20–22
Weight (kg)	72.5 ± 4.8	67.5–77.0
Height (cm)	178.5 ± 3.3	175–182
Body surface area (m^2^)	1.89 ± 0.07	1.82–1.96
BMI	22.8 ± 1.1	21.7–23.9

### 2.2 Emergency rescue clothing (ERC)

**Figure 1:**
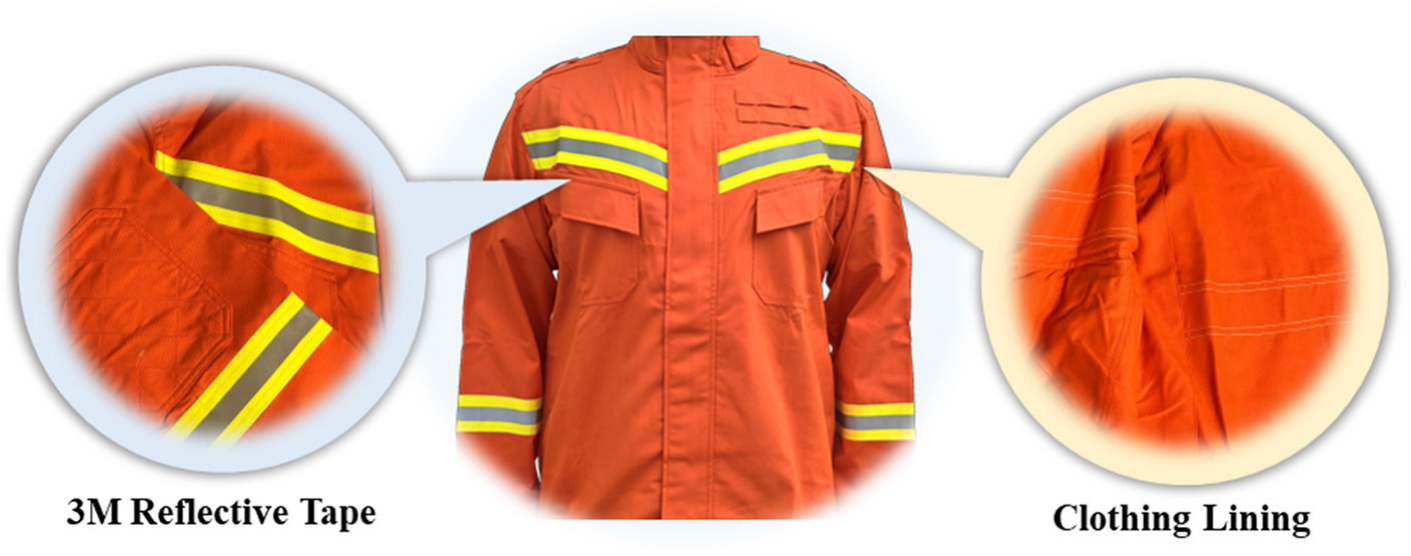
The ERC.

Emergency rescue clothing is in the occurrence of earthquakes, floods, mudslides, traffic accidents and other special environments for emergency rescue personnel to provide effective protection and protection of clothing, with flame retardant, moisture resistance, anti-static and other properties. The ERC (RJF-F1A, Yangzhou Hongan Firefighting Equipment Co., Ltd, China) used in this study is used by Chinese firefighters in environments such as earthquake collapse and flood rescue, complies with GA 633-2006 Firefighters' protective ensemble for rescue. It is a single layered split design with a neck protector at the collar, bright colors and a visible warning (Figure 1). It consists of high-performance meso-aramid fiber with 3M reflective tape, is abrasion-resistant and anti-static, and weighs 600 g for the top and 520 g for the trousers. Using a 20-zone “Newton” sweating thermal manikin, we assessed the total thermal and humidity resistance of ERC in accordance with ASTM F1291-16 and ASTM F2370-16 test protocols (21). The resultant values were 1.5 Clo for thermal resistance and 1.66 m^2^ Pa/W for humidity resistance. The CON group used in this study is a regular pure cotton white T-shirt and black shorts, mainly used as the control group for the experiment. The thermal resistance of the control group was ~0.10 Clo, and the evaporative resistance was estimated at 6 m^2^ Pa/W, based on published thermal manikin data (22). These values reflect typical lightweight summer clothing ensembles.

### 2.3 Experimental setup

**Figure 2:**
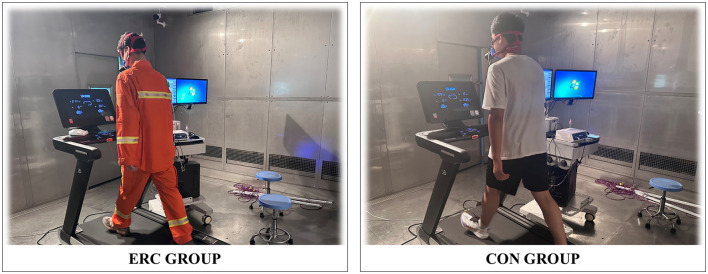
The participant is exercising in the climate chamber.

In an effort to assess the effects of emergency rescue clothing (ERC) on the physiological and psychological stress of emergency responders, we executed two randomized trials: one with emergency rescue clothing (ERC) and the other with conventional attire (CON). Set conditions to simulate a high-temperature and high humidity rescue environment, with a temperature of 35°C and a relative humidity of 75%, as shown in Figure 2.

**Figure 3:**
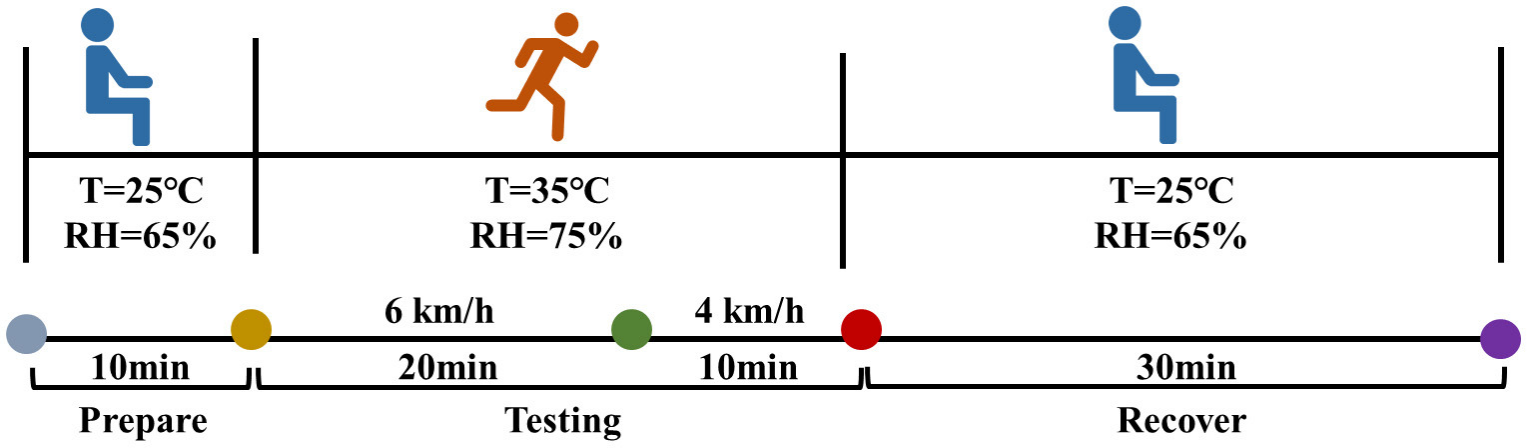
Experimental setup.

This study set up a preparation phase primarily to ensure that participants were able to adapt to the environmental conditions of the climate chamber and stabilize their initial physiological and psychological states. During this period, participants need to rest for 10 min in a climate controlled room with a temperature of 25°C and a relative humidity of 65%. Subsequently, the experimental phase began. During the initial 20 min of the experimental phase, participants were required to walk at a speed of 6 km/h with 35°C and 75% relative humidity, followed by a 10 min walk at a speed of 4 km/h. The average walking speed for adults is about 4.5 km/h (23). After exercise, the subjects returned to the climate chamber at 25°C and 65% relative humidity for 30 min of recovery, as shown in Figure 3. The high-temperature and high-humidity condition (35°C, 75% RH) was selected to simulate typical summer rescue environments in southern China. According to long-term meteorological records, the annual mean relative humidity in Guangdong Province reaches ~78.9% (24), supporting the representativeness of this setting. The control condition (25°C, 65% RH) was chosen to reflect a thermally neutral environment, consistent with benchmark values used in previous field studies (25), and thus serves as a suitable reference for comparative evaluation.

### 2.4 Measurement and calculation methods

Before initiating the trial, participants were informed about the scales for evaluating their testing experience. The test's duration and the intensity of the exercise were fine-tuned in accordance with our research objectives and data from preliminary human trials (26). The trial was halted if participants exhibited any of the following: a core temperature exceeding 39°C, a heart rate surpassing 95% of their predicted maximum, or signs of physiological stress, including confusion or nausea (27).

**Figure 4:**
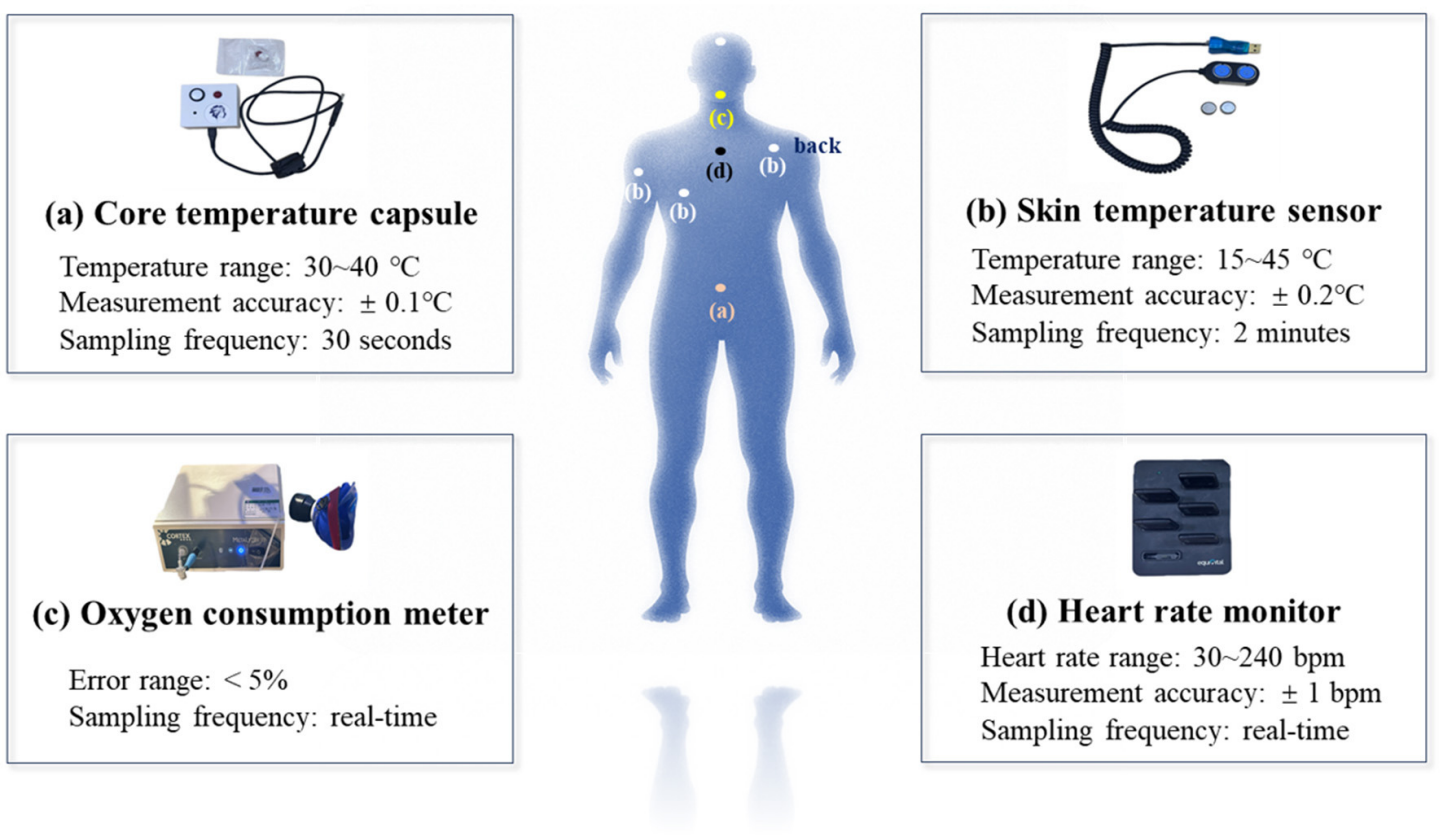
Measurement instruments: **(a)** Core temperature capsule. **(b)** Skin temperature sensor. **(c)** Oxygen consumption meter. **(d)** Heart rate monitor.

Throughout the trial, we meticulously documented a range of physiological responses, including core temperature (*T*_c_), mean skin temperature (*T*_sk_), heart rate (HR), and oxygen consumption. The instruments used to measure physiological parameters are shown in Figure 4. Participants' core temperature (*T*_c_) was measured by taking telemetry pills (CorTemp@, HQInc, Palmetto, USA), which were swallowed ~1 h before the start of the formal experiment. In addition, core temperature was continuously monitored throughout the 10-min rest phase prior to exercise. This allowed us to verify that the capsule had reached a stable position within the gastrointestinal tract and that baseline core temperature values had stabilized before initiating the exercise protocol. This procedure was adopted to account for individual differences in gastrointestinal transit time and to improve data reliability. In this study, All 13 participants' Tc data were successfully collected using the telemetry capsules. Δ*T*_c_ was used instead of absolute Tc to minimize the influence of individual baseline differences and to better reflect dynamic thermoregulatory responses to thermal stress, which is consistent with the standard practice in physiological strain assessments. Specifically, Δ*T*_c_(*t*) = *T*_c_(*t*)–*T*_c0_, where *T*_c0_ represents the average of the last 2 min of core temperature during the rest phase, and *T*_c_ (*t*) denotes the core temperature at a given time point *t*. The temperature measurement accuracy is ± 0.1°C, and a sample is collected every 30 s. Oxygen consumption was also recorded during the experiment. The oxygen consumption measurement device can simultaneously measure parameters such as metabolic equivalent, carbon dioxide emission, and respiratory exchange ratio, with an error range typically < 5%. To capture rapid changes in breathing rhythm, the sampling interval is set to record data in real time. By attaching sensors to the forehead, left chest, left upper arm, and back, participants' skin temperature (*T*_sk_). The mean skin temperature was calculated using a weighted formula with coefficients of 0.28, 0.28, 0.16, and 0.28, respectively, which is an adaptation of the four-point model originally proposed by Ramanathan for estimating surface temperature distribution of the human body (28). The skin temperature sensor has an error margin of ~± 0.2°C, and data is received every 2 min. Participants' heart rates were measured using heart rate monitors. These devices typically have an accuracy of ± 1 bpm and a sampling frequency of 256 Hz, ensuring precise tracking of dynamic heart rate fluctuations.

The PSI as depicted in Equation 1 (29), quantifies the level of physiological stress experienced by individuals. It is a numerical scale that spans from 1 to 10, categorized into three distinct levels: none-mild (0–2.9), low-moderate (3.0–6.9), and high-very high (7.0–10).


(1) $$\begin{array}{c} PSI=\frac{5\times \left(T_{c}-T_{C0}\right)}{39.5-T_{c0}}+\frac{5\times \left(HR-60\right)}{HR_{\max}-60} \end{array}$$

where *T*_c_ (°C) as the core temperature and HR (bpm) as the heart rate, both measured simultaneously at any specific point in time; *T*_c0_ represents the core temperature at rest (°C); HRmax denotes the maximum attainable heart rate (bpm).

**Figure 5:**
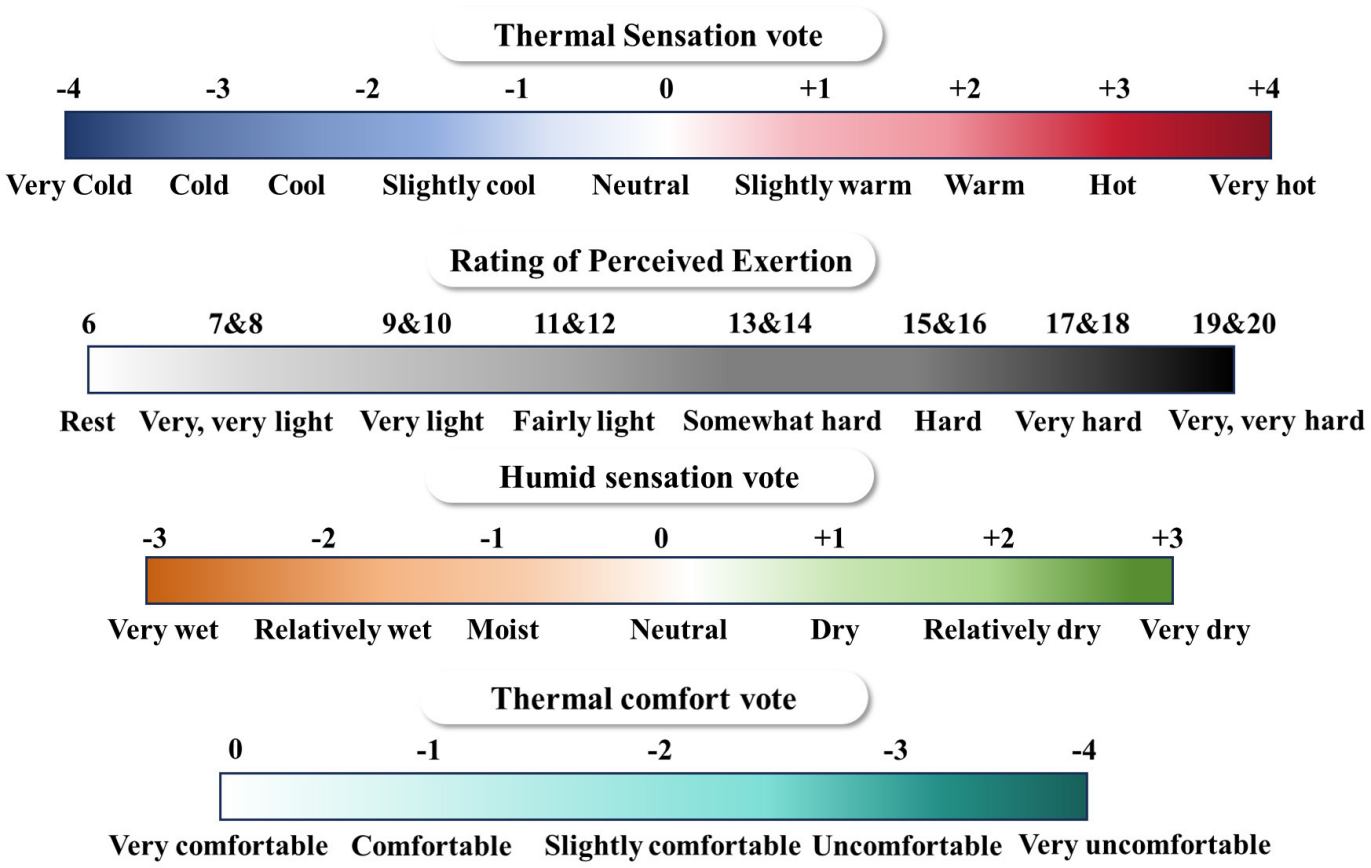
Participant evaluation scales.

In addition, this study also measured the perceptual parameters of the subjects through questionnaire surveys during the preparation phase, experimental phase and recovery phase, including thermal sensation vote (TSV), thermal comfort vote (TCV), rate of perceived exertion (RPE), and humid sensation vote (HSV), with results recorded every 5 min. To ensure data accuracy and participant safety, a paper questionnaire was filled out every 5 min during the experiment, and participants conducted subjective evaluations while in a stationary state. Using a nine-point scale to measure thermal sensation (30). Humid sensation vote was judged by the degree of warmth of the skin. Thermal comfort is the subjective satisfaction of the human body with its thermal environment. RPE was recorded using a subjective rating scale of 15 points, ranging from 6 to 20 (31). The specific scales used for voting are shown in Figure 5.

Equation 2 is obtained based on Tiguisis' 0–19 scale PeSI (32).


(2) $$\begin{array}{c} PeSI=\frac{5+\left(TSV+3\right)}{6}+\frac{5\times \left(RPE-6\right)}{14} \end{array}$$

where TSV and RPE were recorded at various time points during the experiment.

### 2.5 Data processing

To comprehensively evaluate the physiological and psychological responses of participants, we measured key indicators such as *T*_c_, *T*_sk_, HR, and oxygen consumption, and subjectively assessed TSV, TCV, RPE, and HSV. These data were calculated using mean and standard deviation, and statistical significance was established when the *p*-Value < 0.05. Subsequently, SPSS was used for one-way analysis of variance (ANOVA) to determine significant differences between the two groups. If there is a significant difference in the experimental results between the two sets, it will be indicated by an asterisk (^*^) in the image. In addition, correlation analysis was conducted using Origin software to calculate the relationship between physiological and psychological parameters. The *R*-value is used to indicate the strength of the association. The range of values for the correlation coefficient is −1 to 1, where 0 indicates no relationship, 1 indicates complete positive correlation, and −1 indicates complete negative correlation.

## 3 Results

### 3.1 Physiological responses

#### 3.1.1 Core temperature

**Figure 6:**
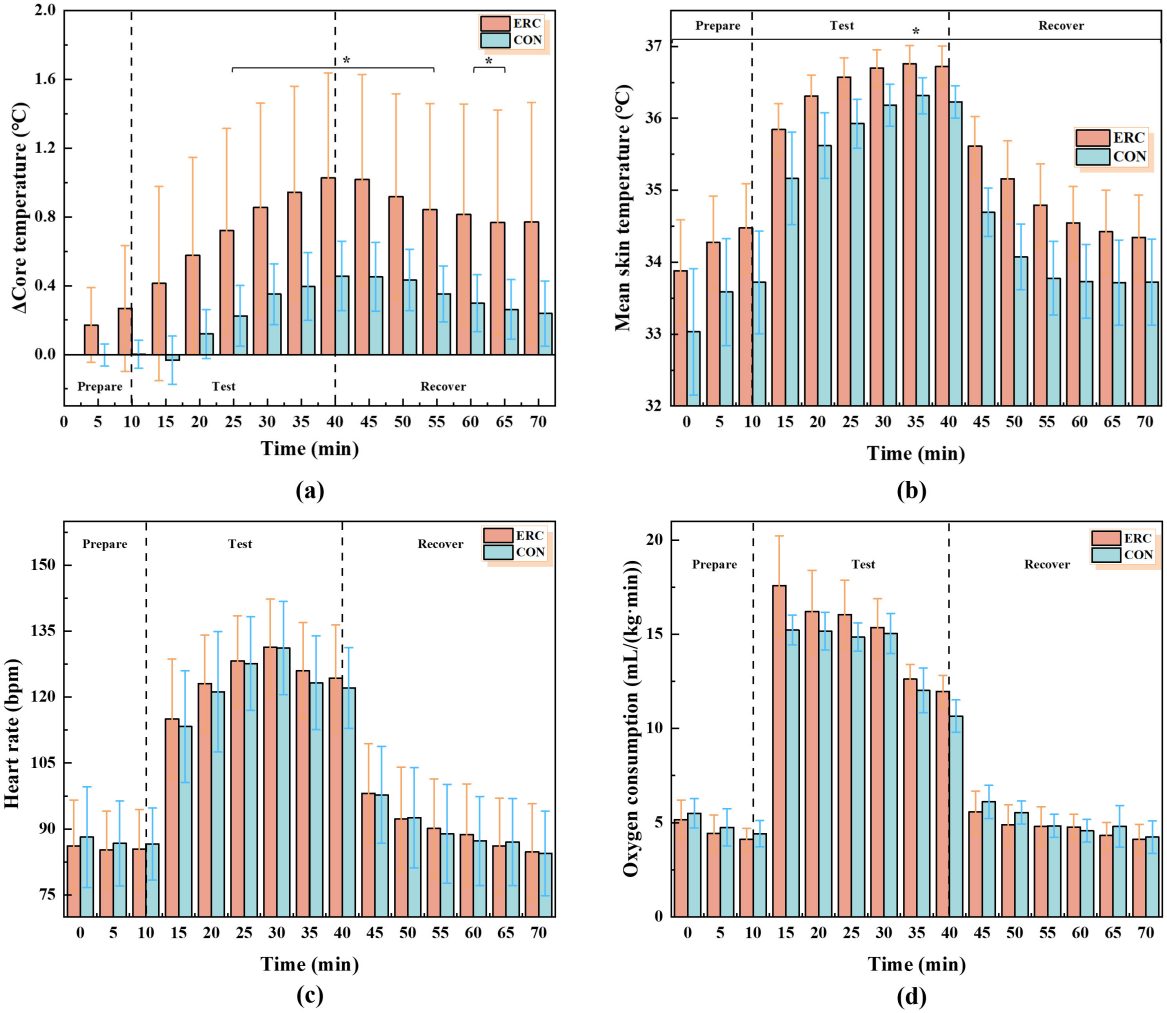
Physiological parameter images: **(a)** Δ Core temperature in ERC and CON conditions. **(b)** Mean skin temperature in ERC and CON conditions. **(c)** Heart rate in ERC and CON conditions. **(d)** Oxygen consumption in ERC and CON conditions.

Figure 6 shows the physiological parameters of the subjects, including core temperature, average skin temperature, heart rate, and oxygen consumption. The trend of Δ*T*_c_ in the two groups of experiments is shown in Figure 6a. The overall trend of Δ*T*_c_ in the ERC group and the CON group is a gradual increase in the test stage and a slow decrease in the recovery stage. The maximum values of Δ*T*_c_ in the ERC group and the CON group are 1.03 and 0.46°C, respectively, and both appear at the 40th min of the experiment, which is the moment when the subject ends the exercise. Compared to the initial value, this has increased by 0.86°C (ERC) and 0.46°C (CON), respectively. In the intervals of 25–55 and 60–65 min, there are significant differences between the two test groups (*p* < 0.05), with a maximum difference of 0.57°C. This indicates that wearing ERC has a certain impact on the core temperature of the subjects during exercise. Even after sitting quietly for 30 min in a room temperature environment of 25°C, the core temperatures of the two groups of experiments have not returned to the state before exercise.

#### 3.1.2 Mean skin temperature

During the exercise phase, there was a notable elevation in mean skin temperature, which subsequently experienced a swift decline during the recovery period. As shown in the Figure 6b, the mean skin temperature of the ERC group was higher than that of the CON group. The highest mean skin temperature of the ERC group and CON group were 36.75 and 36.31°C, respectively, which increased by 2.87 and 3.28°C compared to the initial values. There was a significant difference between the ERC group and the CON group from 0 to 70 min, with the maximum difference being 1.08°C. These differences in skin temperature may affect the comfort level of the subjects, and higher skin temperatures when wearing emergency rescue suits may make people feel stuffy and uncomfortable. Upon conclusion of the experiment, the ERC group exhibited a mean skin temperature of 34.34°C, while the CON group registered 33.72°C. The disparity in these final measurements from their initial values suggests that the recovery period was inadequate for a full return to baseline skin temperature.

**Figure 7:**
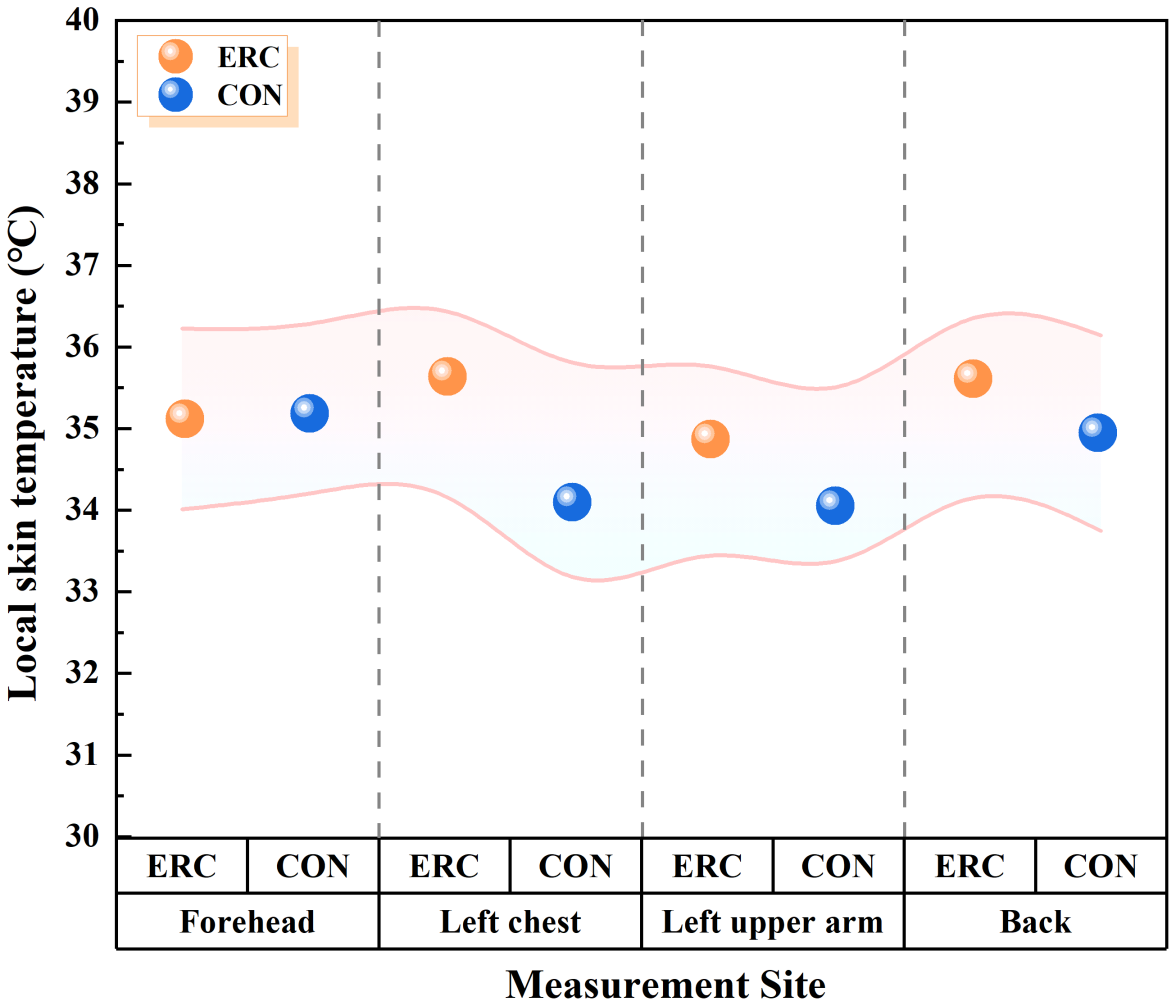
Local mean skin temperature of subjects wearing ERC and CON.

Figure 7 shows the local skin temperature during the experiment (70 min) while wearing ERC and CON. Except for the forehead area, the mean skin temperature of the left chest, left upper arm, and back of the subjects wearing ERC was higher than that of CON. The mean skin temperatures on the forehead were 35.11 and 35.19°C (ERC and CON), the mean skin temperatures on the left chest were 35.64 and 34.10°C (ERC and CON), the average skin temperatures on the left upper arm were 34.87 and 34.05°C (ERC and CON), and the average skin temperatures on the back were 35.60 and 34.95°C (ERC and CON), respectively.

In this figure, “bands” refer to the shaded areas or error bars representing the standard deviation (or standard error) of skin temperature at each sensor location. These are used to illustrate the variability of measurements among participants.

#### 3.1.3 Heart rate

The heart rate trends of the two sets of trials are shown in Figure 6c. The heart rate curves of the ERC and CON groups showed an identical trend, with no significant difference between the two groups. However, we can observe that both groups reached their peak at the 30th min of the experiment, at which point the maximum heart rate in the ERC group was 131.33 bpm, and the maximum heart rate in the CON group was 131.14 bpm. The peak reached at the 30th min is related to the setting of this experiment, where the subject's walking speed is 6 km/h and the amount of exercise is maximum. The increase in heart rate during exercise is consistent with standard cardiovascular responses to physical exertion. Therefore, when exercising for about 20 min, the cumulative effect of these factors leads to a peak in heart rate.

#### 3.1.4 Oxygen consumption

During the preparation phase (0–10 min), the CON group consumed more oxygen than the ERC group; during the exercise phase (15–40 min), the ERC group consumed more oxygen than the CON group; and during the recovery phase (45–70 min), the CON group consumed more oxygen than the ERC group overall, with a change only at 60 min. Between the two groups of tests, significant differences were seen at 15 and 40 min, when the differences were 2.35, 1.30 ml/(kg·min). In both cases, the peak was reached at the 15th min of the test, 17.57 ml/(kg·min) in the ERC group and 15.22 ml/(kg·min) in the CON group.

#### 3.1.5 PSI

**Figure 8:**
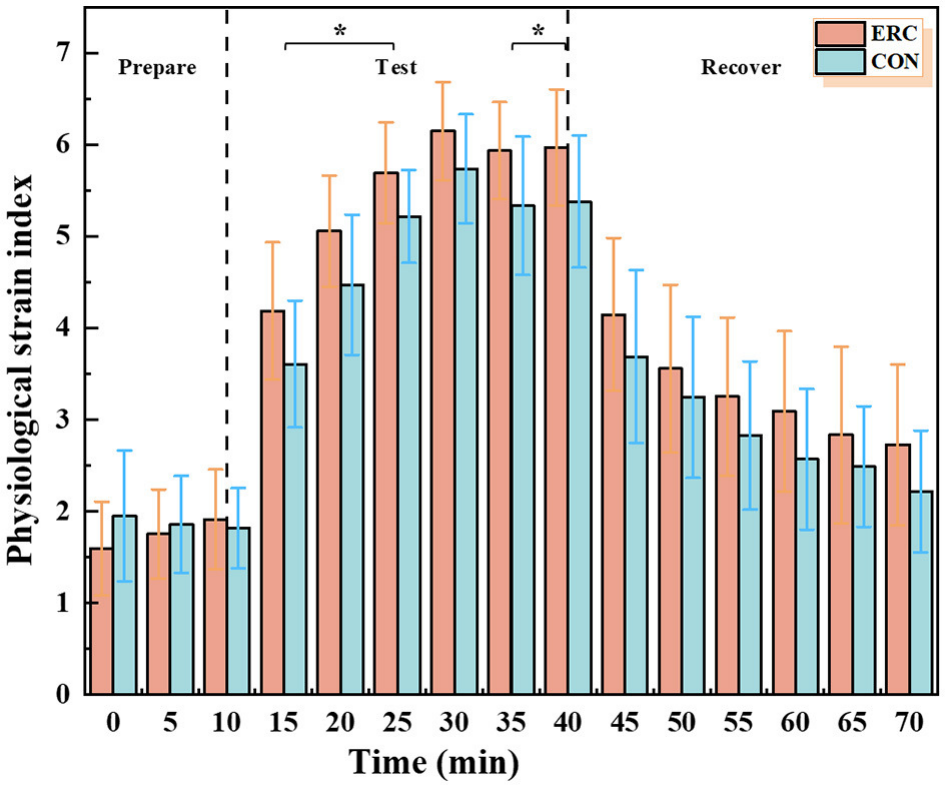
PSI in ERC and CON conditions.

The trends in PSI for both sets of trials are shown in Figure 8. The PSI in the ERC and CON groups reached a maximum value of 6.15 and 5.73, respectively, at the 30th min of the experiment. In both cases, participants felt a “no-little” physiological stress in the initial stage, a “moderate” physiological stress level in the experimental stage, and then slowly decreased, but did not recover to the level of the initial preparation stage until the end of the experiment. Significant differences were observed in PSI between the 15–25 and 35–40 min ranges.

### 3.2 Perceptual responses

#### 3.2.1 Thermal sensation vote

**Figure 9:**
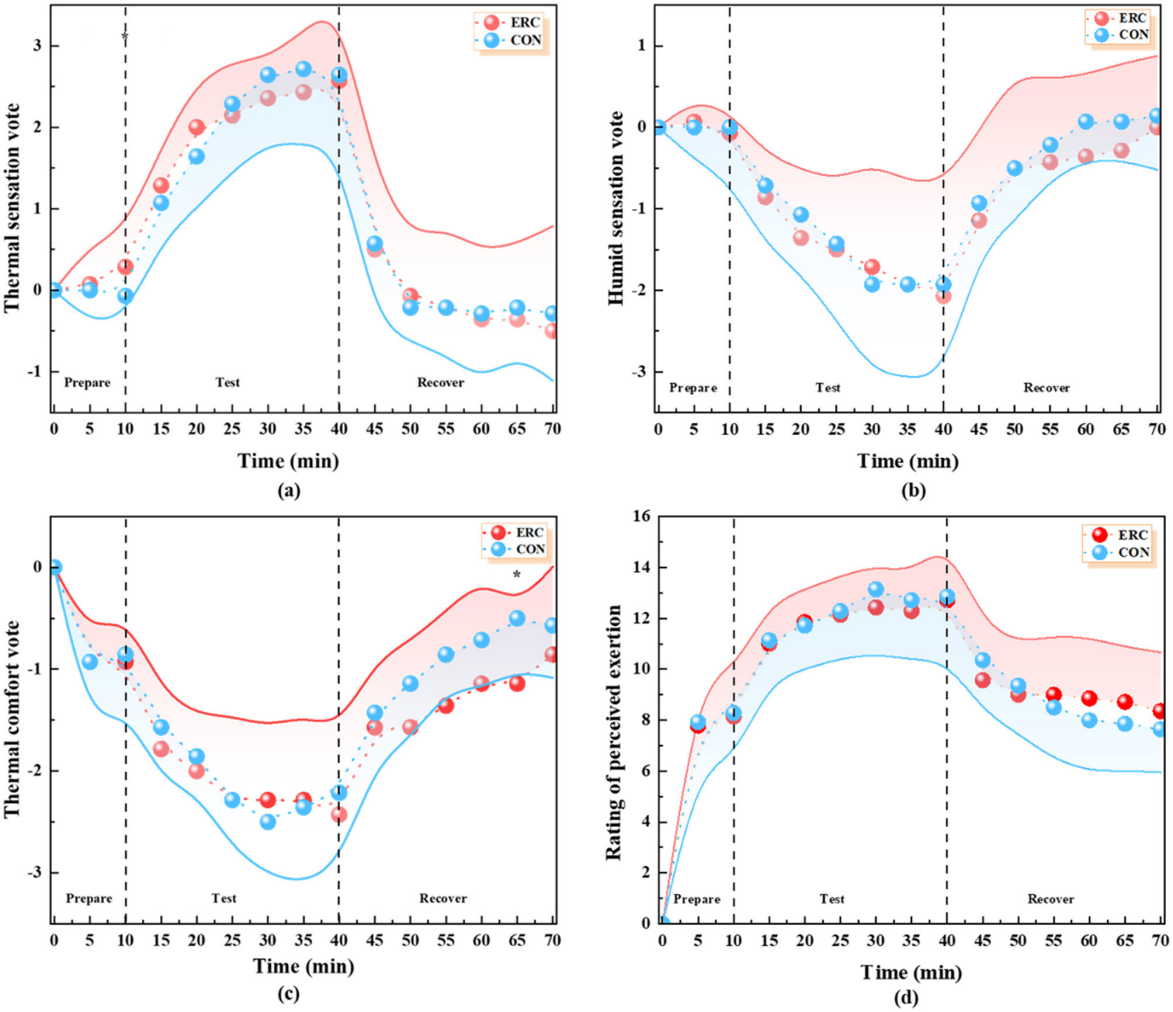
Perceptual parameter images: **(a)** TSV in ERC and CON conditions. **(b)** HSV in ERC and CON conditions. **(c)** TCV in ERC and CON conditions. **(d)** RPE in ERC and CON conditions.

There was a significant difference between the ERC group and the CON group only at the 10th min, as shown in the Figure 9a. At this time, the TSV of the ERC group was 0.46 scales, while the CON group was 0.28 scales. The maximum difference between the two groups was 0.18 scales. Research has shown that in both sets of tests, except for the 10th min, ERC had no significant effect on human thermal sensation.

#### 3.2.2 Humid sensation vote

As shown in the Figure 9b, the curves of the two sets of data showed an identical trend. The HSV of the ERC group was the same as that of the CON experimental group at 0-, 35th, and 50th min. At this time, the humid sensation scores of the subjects were 0, −1.92, and −0.5, respectively. According to the SPSS data, there was no significant difference in HSV between the two groups of tests, indicating that the impact of clothing on human humid sensation was not significant.

#### 3.2.3 Thermal comfort vote

During the resting phase, participants reported feeling either “comfortable” or “just comfortable,” transitioning to “uncomfortable” or “very uncomfortable” during the exercise phase, and then returning to a normal comfort level by the end of the experiment's resting phase, as shown in the Figure 9c. Although the difference in thermal comfort perception between the ERC and CON groups was not statistically significant, the maximum discrepancy observed at the 65-min mark was 0.64. The difference in TCV at the 65th min may be related to the environment in which the subject is currently located. In the early stages of the experiment, participants' attention may be more focused on the exercise itself or adapting to the environment, while in the recovery stage, when the body is in a relatively relaxed resting state, participants may pay more attention to their personal feelings, especially in terms of TCV. At this point, the differences caused by wearing different clothing are more easily noticed, leading to differences in TCV at the 65th min.

#### 3.2.4 Rate of perceived exertion

As illustrated in Figure 9d, no statistically significant differences were observed in RPE between CON group and ERC group across trials. This result indicates a comparable level of perceived physical strain in both conditions. All subjects felt “very relaxed or relaxed” during the preparation and recovery phases, and “slightly stretched” during the test phase. Notably, the maximum in the ERC group occurred at 40th min, when the RPE level was 12.71, and the maximum in the CON group occurred at 30th min, when the RPE level was 13.14.

#### 3.2.5 PeSI

**Figure 10:**
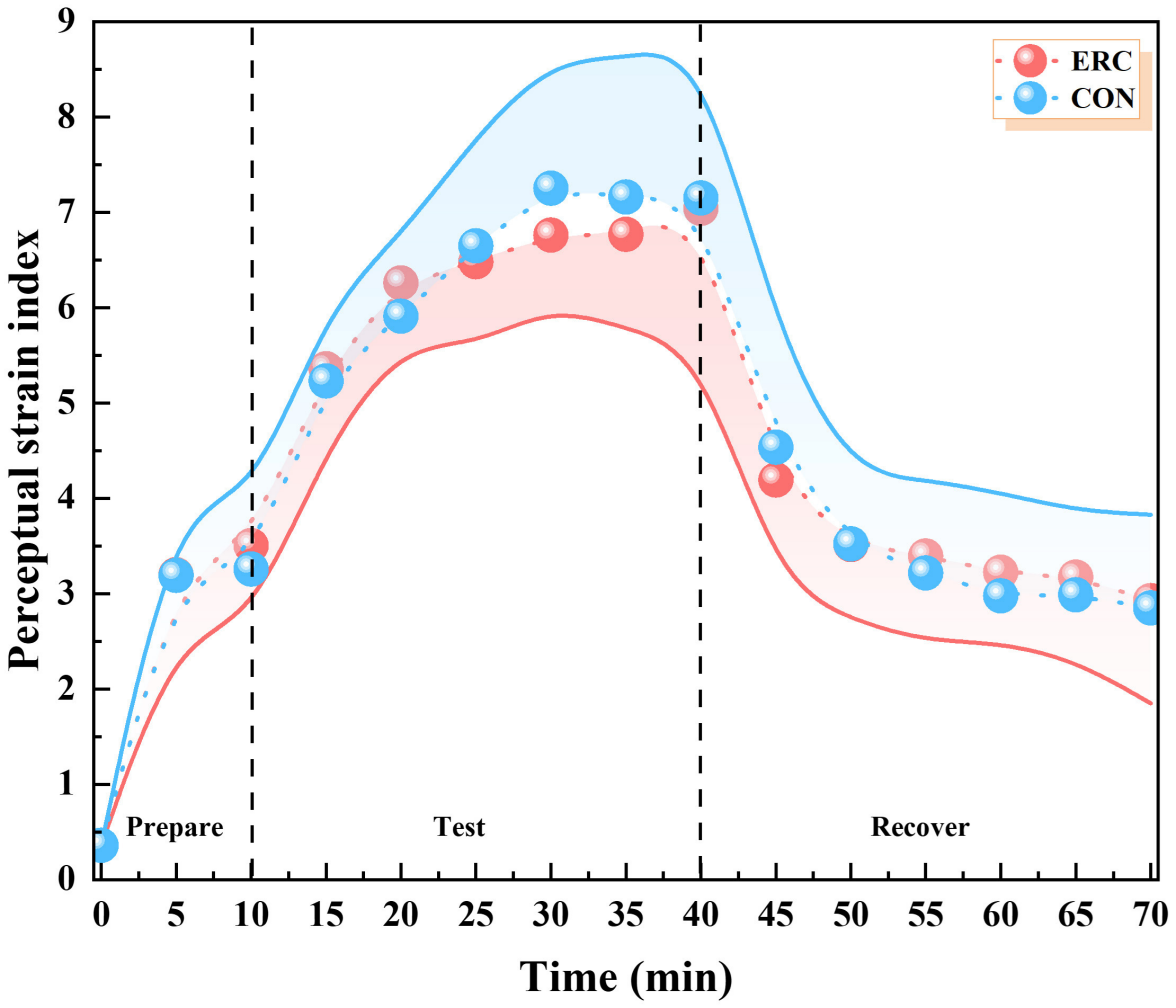
PeSI in ERC and CON conditions.

As shown in the Figure 10, the PeSI for both CON and ERC groups exhibited a pattern of initial increase followed by a decrease. At 0–40 min, PeSI rose in tandem with the progression of time, peaking at levels categorized as “high” or “very high” with values of 7.04 for the ERC group and 7.25 for the CON group. In the subsequent recovery phase, PeSI progressively declined for both groups. Ultimately, PeSI returned near to baseline levels, with 2.92 for the ERC group and 2.85 for the CON group, closely aligning with the pre-exercise resting values of 3.20 for both groups. Throughout the 70-min experimental trial, the variance in PeSI between the two groups did not achieve statistical significance.

## 4 Discussion

### 4.1 Effect of ERC on physiological responses

Core temperature serves as a critical metric for assessing thermal strain on the human body (33). An elevation in core temperature can impair the body's thermoregulatory capabilities and diminish physical performance. Concurrently, it predisposes individuals to fatigue and poses potential, health hazards (34). In this study, the overall trend of Δ*T*_c_ in the ERC group and the CON group is a gradual increase in the test stage and a slow decrease in the recovery stage. In the test stage, the body heat production of the subjects is greater than the heat dissipation, and the heat accumulates in the body. As the exercise continues, the core temperature gradually rises. After entering the recovery stage, the subject's body activates the heat dissipation mechanism, and the core temperature slowly decreases. As the subjects moved from the exercise phase to the recovery phase, differences between the ERC and CON groups began to emerge. This may be related to the stabilization of the physical state of the subjects as they acclimatize to the ambient temperature. The overall core temperatures in the ERC group are basically slightly higher than in the CON group. This demonstrates a certain thermal strain response in subjects wearing PPE. However, there was little change in core temperature between the ERC and CON groups, which may be related to the thermal resistance of the ERC. Under the environmental conditions of air temperature of 21°C, relative humidity of 50% and wind speed of < 0.1m/s, the thermal resistance of the clothing worn by people who are sitting quietly or engaged in light mental labor (the metabolic rate of the human body is 58.1 W/m^2^) is 1 Clo when they feel comfortable (35). In this study, the thermal resistance of the ERC was calculated to be 1.5 Clo by means of thermal manikin experiments. For the human body, this value perceives a higher degree of thermal comfort in the garment. This is consistent with results reported by (36), which showed that clothing plays an important role in the body's thermal balance. The effect of clothing thermal resistance on human thermal comfort is more pronounced.

Mean skin temperature is a pivotal parameter in predicting thermal sensation and comfort in humans, as it directly influences the convective and radiative heat exchange processes between the human body and the surrounding environment (37). This indicator is crucial for assessing the efficacy of personal protective equipment and the design of indoor environments to ensure optimal thermal conditions for occupants (38). Typically, the thermal and moisture resistance of a garment is the most fundamental component in assessing the comfort of a garment (39). However, in different climatic environments and levels of human activity, the body wears clothing that produces different levels of heat and moisture exchange, which affects the body's metabolic rate level (40). In this study, the subjects' skin temperature did not return to the initial level from the beginning to the end. Mean skin temperature increased by 2.87 and 3.28°C in the ERC and CON groups, respectively. It was demonstrated that after 30 min of exercise in an environment with a temperature of 35°C and 75% relative humidity, only 30 min of recovery did not allow personnel to rest adequately. However, about 10 min or so after the personnel entered the recovery phase, the mean skin temperatures of both the ERC and CON groups showed a tendency toward a steady decline.

In addition, by observing the mean skin temperature of different parts, we believe that this temperature change may be related to the material properties of the clothing. The emergency rescue suit with aramid fiber structure is relatively tight. Although this helps to improve the strength and durability of clothing, it limits air circulation and results in poor breathability. The moisture and heat on the skin surface of subjects wearing first aid suits are difficult to dissipate, resulting in higher average skin temperatures on the left chest, left upper arm, and back (41). Moreover, the frontal area was not covered by any clothing, so it was observed that the frontal temperatures of the ERC group and CON group were very close. However, when wearing the CON group, the forehead temperature of the subjects was higher than that of the ERC group. T-shirts and short sleeves are usually lightweight and breathable, making them easier to dissipate body heat. But this heat dissipation may not be uniform. Due to the abundant blood vessels in the frontal region and its proximity to the brain, which is a high heat producing organ, when the overall heat dissipation of the body is good, the blood will bring more heat to the frontal region, resulting in a relatively high temperature in the forehead. Although emergency rescue suits hinder the heat dissipation of other parts of the body, they can regulate the distribution of heat in the body, allowing more heat to be conducted or dispersed to other parts, thereby relatively reducing the temperature in the forehead area (42).

Changes in heart rate are closely related to heart disease and are an important indicator of cardiovascular response. The ratio of maximum heart rate (220 bpm-Age) was 66.37% (131.33 bpm) and 65.53% (131.14 bpm) in the ERC and CON groups, respectively. Based on the categorization of heart rate as a percentage of maximum values (43), the participants exhibited moderate cardiovascular strain. The similarity in heart rate responses between the two trial groups was not statistically significant, a result that corroborates the research conducted by (44). Heart rates varied widely among the study subjects and may have been caused by a number of factors. Heart rate peaked at the 30th min of the experiment in both the ERC and CON groups. At this point was the peak of the subject's high-intensity training at 6 km/h in an environment with a temperature of 35°C and 75% relative humidity. The subjects started exercising at a speed of 4 km/h, and subsequently the subjects' heart rate started to show a decrease. This demonstrates the ability of heart rate to respond to a subject's physical state in real time. In the future firefighters carrying out rescue work, heart rate is a parameter that should continue to be monitored as an important indicator of the safety of firefighters' lives. In this study, mean skin temperature showed a positive correlation with heart rate, which is in line with previous studies (45).

Metabolic rate plays a crucial role in the body's heat balance and temperature regulation, the maximum value of oxygen consumption during the trials was observed at the 15th min. Moreover, ERC group significantly higher than the CON group. This is consistent with previous research (46), which found that wearing PPE typically leads to a 20%−45% increase in oxygen consumption in the human body. The peak at the 15th min may be related to the intensity of exercise. In the early stages of exercise, the body primarily relies on aerobic metabolism to provide energy. When the subject transitions from a resting state to an active state, the body's energy demand rapidly increases. In the first few minutes of exercise, the body needs to quickly adjust to meet the increased energy demand, resulting in a sharp increase in oxygen consumption. As exercise continues, the body gradually adapts to the current intensity of exercise and various physiological functions reach a relatively stable state. Therefore, it was observed that the oxygen consumption of the subjects gradually stabilized after 20–30 min of the experiment.

During the experimental phase, the ERC and CON groups exhibited “moderate” levels of physiological strain, with PSI scales of 6.15 and 5.73, respectively. After 30 min of recovery, these scales reached to “none-little” levels, with the PSI of the ERC group dropping to 2.73 and the CON group dropping to 2.22. There was a significant difference between the two types of clothing between the 15th and 25th min of the experiment. This indicates that although ERC may increase physiological stress, the impact is relatively small.

### 4.2 Effect of ERC on perceptual responses

While previous studies have incorporated cognitive function assessments such as decision-making tasks or reaction time tests under heat stress, these measures often require specialized equipment, controlled laboratory conditions, or interruptive protocols that may not be practical in field settings. In contrast, our study prioritizes perceptual indices—such as TSV, TCV, RPE and the PeSI, which have demonstrated value in operational environments due to their simplicity, rapid implementation, and close alignment with subjective experience. These indices provide real-time insights into how individuals perceive thermal load and exertion, making them especially useful for evaluating emergency protective gear in high-stress conditions. By focusing on these perceptual parameters, our research contributes a complementary perspective to existing literature, bridging physiological responses and field-relevant subjective evaluation.

RPE can indicate the sensitivity of exercise intensity to cardiovascular and thermoregulatory loads (47). Throughout the experiment, there was no significant difference in RPE between the two groups. The time when subjects in the ERC and CON groups felt most fatigued occurred at the 40th and 30th min, respectively. The peak values of ERC and CON groups appearing at different time points may be related to the different fatigue accumulation rates caused by clothing characteristics. Although wearing emergency rescue clothing hinders the dissipation of heat, to a certain extent, clothing provides more physical support and protection, making the accumulation of fatigue relatively slow. T-shirts and shorts can better promote heat dissipation in high temperature and high humidity environments, allowing the body to dissipate the heat generated during exercise relatively quickly, thereby delaying the accumulation of fatigue. However, as exercise continues, even with good heat dissipation, by 30 min, factors such as energy expenditure, accumulation of metabolites, and slight muscle damage will gradually lead to a peak in fatigue levels. In addition, there is a positive correlation between the weight of clothing and fatigue. In this study, the weight of the ERC was only 1.5% of the subjects' body weight. As a result, the ERC did not cause excessive fatigue throughout the trial. RPE is also related to the time and intensity with which a person unfolds their work. In this study, there was an effect of 30 min of exercise intensity on the skin temperature of the personnel. However, from the RPE scales, it has not been observed that exercising at 6 km/h for 20 min or 4 km/h for 10 min makes the human body feel “very strenuous,” but only “slightly strenuous” at present.

TCV and TSV reflect participants' subjective thermal perceptions during the trials. TCV represents overall comfort, while TSV indicates perceived heat intensity. In our study, no statistically significant difference in TCV was observed between the ERC and CON groups, suggesting that although ERC increased core and skin temperatures, it did not substantially alter participants' subjective comfort levels. This may be attributed to the short exposure duration, as well as the participants' young age and physical fitness, which could enhance thermal tolerance.

For TSV, participants in both groups reported increased thermal sensation between min 15 and 40, corresponding to the exercise period under high heat and humidity. This increase aligns with elevated skin and core temperatures and the onset of thermoregulatory responses such as sweating. However, the lack of significant group differences in TSV suggests that despite the higher thermal resistance of the ERC, the subjective heat perception was not drastically worsened compared to the lightweight CON clothing. This may imply that perceptual adaptation or clothing design features (e.g., breathability, fit) mitigated some discomfort, which warrants further investigation in longer or task-specific scenarios.

### 4.3 PeSI, PSI and correlation analysis

Correlation analysis is a method of studying whether there is a quantitative correlation between two or more variables. It measures the strength and direction of relationships between variables by calculating correlation coefficients. The range of values for the correlation coefficient is −1 to 1, where 0 indicates no relationship, 1 indicates complete positive correlation, and −1 indicates complete negative correlation. In this study, correlation analysis was applied to explore the direct relationship between physiological parameters and perceptual parameters.

#### 4.3.1 The relationship between thermal comfort vote and physiological parameters

**Figure 11:**
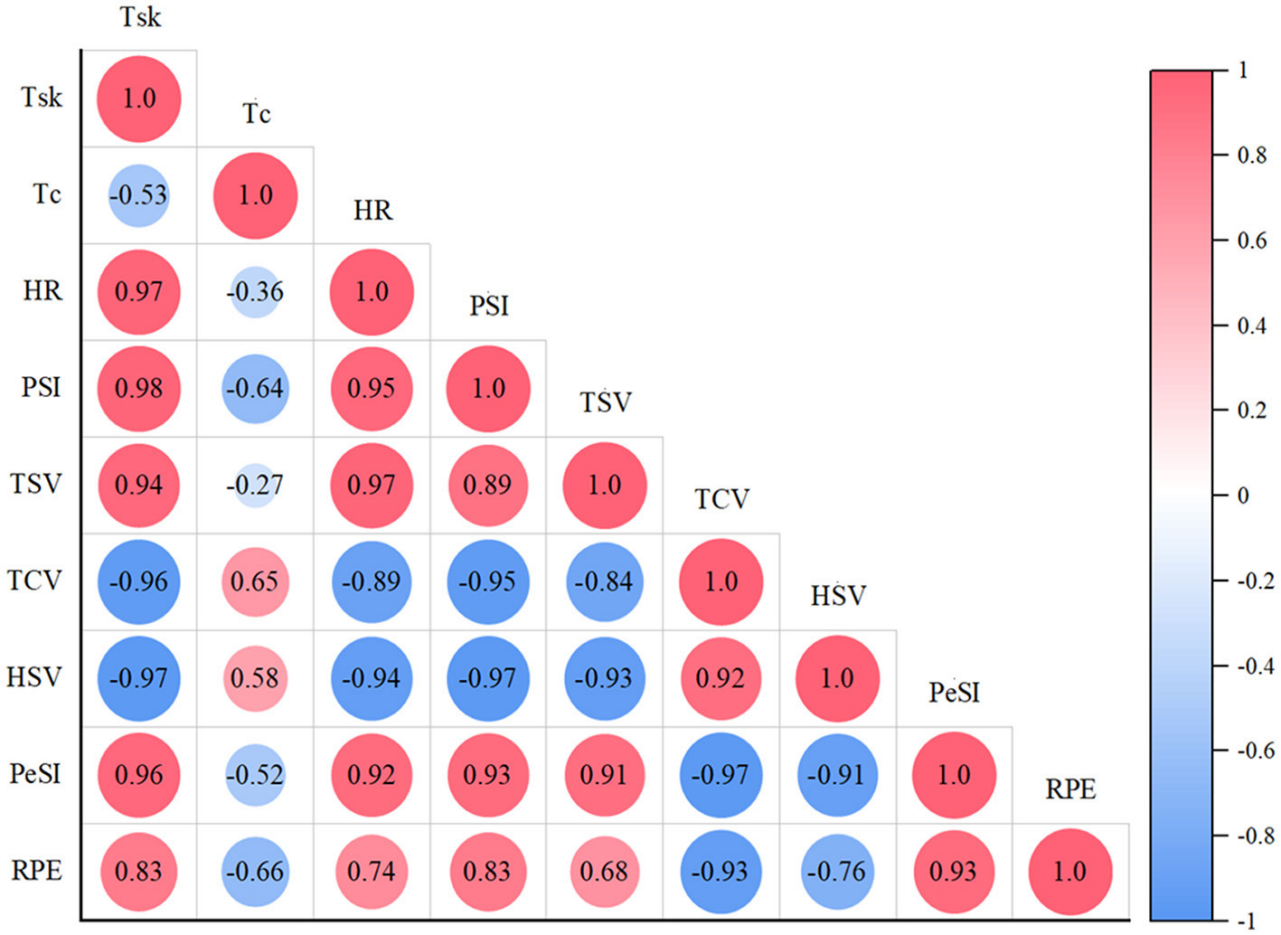
Correlation diagram of various parameters in ERC group.

**Figure 12:**
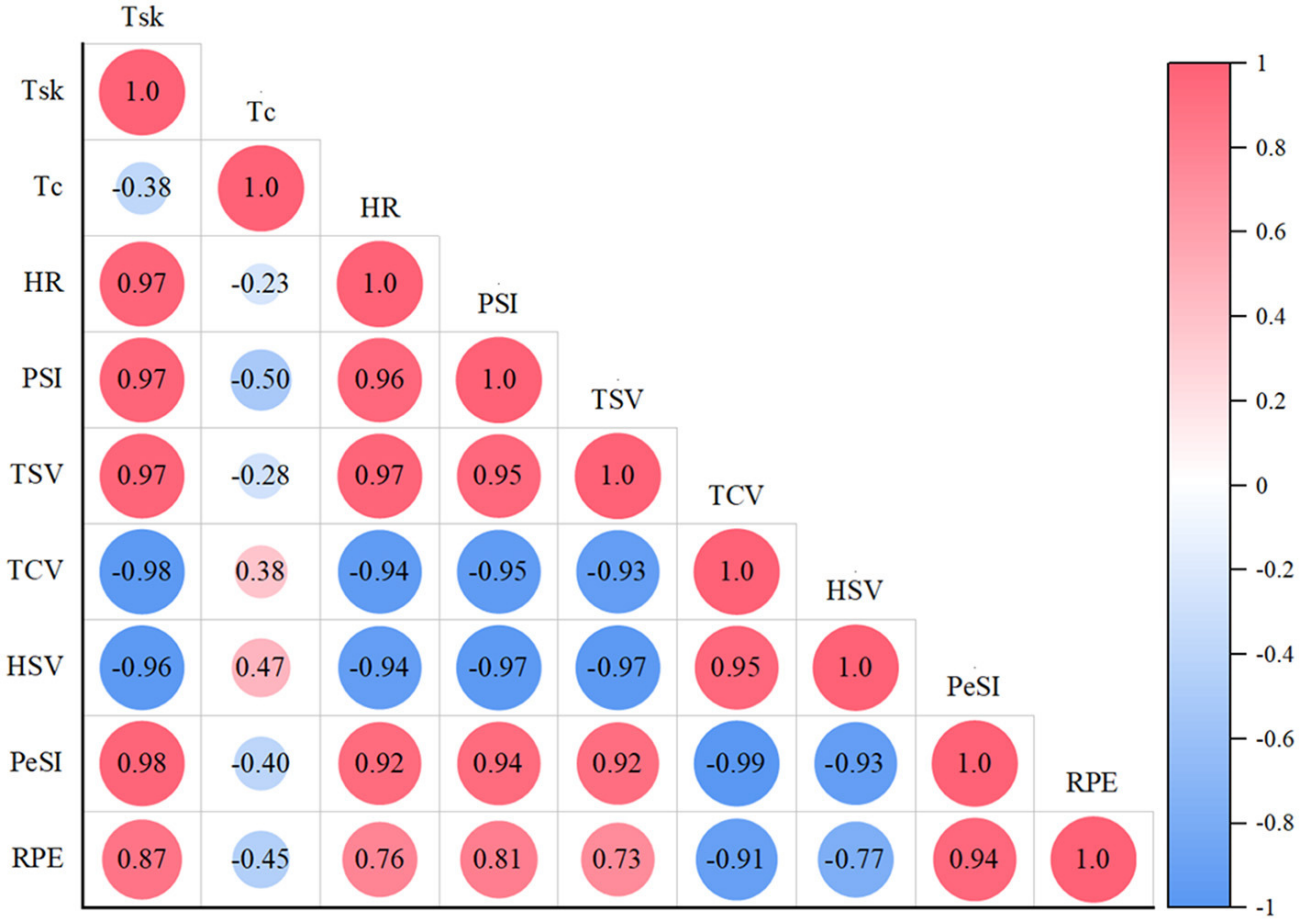
Correlation diagram of various parameters in CON group.

Correlation analysis shows that there is a strong negative correlation between TCV and PSI, *T*_sk_, HR in both sets of experiments, as shown in Figures 11, 12. The *R* values for the ERC group and CON group were −0.95 (−0.95), −0.96 (−0.98), and −0.89 (−0.94), respectively. This means that when the thermal comfort level of the human body decreases, the values of PSI, *T*_sk_, and HR usually increase, and vice versa. It can be seen that TCV can effectively reflect an individual's physiological response in a hot environment, and a decrease in thermal comfort usually indicates an increase in physiological load, which can be used as an indicator to evaluate physiological strain levels.

#### 4.3.2 The relationship between thermal sensation vote and physiological parameters

Under two different clothing conditions, significant positive correlations were observed between TSV and HR, *T*_sk_, and PSI (ERC group: TSV and HR were 0.97, TSV and *T*_sk_ were 0.94, TSV and PSI were 0.89; CON group: TSV and HR are 0.97, TSV and *T*_sk_ are 0.97, TSV and PSI are 0.95). This result indicates that thermal sensation can be used to predict changes in heart rate, skin temperature, and physiological strain index. As the thermal sensation increases, the individual's heart rate, skin temperature, and physiological strain index also rise, indicating a strong relationship between thermal sensation and physiological parameters, and the effect of thermal sensation is similar under different clothing conditions. When the human body is in different environmental conditions, the correlation between core temperature and thermal sensation is weak. Among them, metabolic rate can characterize the core temperature of the human body, and skin temperature can characterize the thermal sensation of the human body (48).

#### 4.3.3 The relationship between PeSI and PSI

PeSI can reliably and effectively evaluate human thermal strain (25), without the risk of inaccurate errors in complex operations and equipment use (e.g., pills used to measure core temperature). Throughout the 70-min experimental trial, the two groups did not achieve statistical significance in PeSI. This means that although the emergency rescue clothing is functionally different from T-shirts and shorts, it does not bring too much restraint, pressure, or insecurity to the subjects during the actual wearing process. Therefore, the impact of psychological pressure on subjects such as heat perception and fatigue level are similar.

The positive association between PeSI and PSI was substantiated, with *R* of 0.93 for the ERC group and 0.94 for the CON group. This strong positive correlation indicates that in high temperature and high humidity environments, an increase in psychological stress is often accompanied by an increase in physiological stress. Psychological stress may enhance physiological stress responses, such as increased heart rate and skin temperature, by activating the autonomic nervous system and stimulating sympathetic nervous system. This result indicates that individuals not only have to cope with the potential adverse effects of heat stress in high temperature environments, but also experience significant psychological pressure, thereby further increasing physiological stress responses. Therefore, the positive correlation between PeSI and PSI reveals the interaction and superposition effects of psychological and physiological stress responses. This study enable replace the physiological indicators (*T*_sk_, *T*_c_, HR), which are complex and cumbersome to characterize, by calculating perceptual indicators (TSV, TCV, HSV, RPE) for the emergency responders who carry out their work in the field. Due to the potential limitations of continuous monitoring of core temperature and heart rate during on-site tasks, subjective evaluation can serve as a convenient alternative, but should be supplemented with physiological parameter validation when conditions permit. Although PeSI can be used for rapid assessment, core temperature (*T*_c_) remains an irreplaceable early warning indicator for heat stress, especially when used in conjunction with sustained high temperature tasks.

The findings of this study suggest several directions for improving the design of emergency rescue clothing. Future PPE could integrate lightweight, high-breathability fabrics and smart textiles that enhance moisture management and thermal comfort without compromising protective performance. Modular designs that allow responders to adjust layers according to heat stress levels may also be beneficial in reducing physiological strain during rescue operations.

### 4.4 Limitations of this study

The findings of this study offer valuable insights into the thermoregulatory mechanisms and subjective comfort perceptions of individuals wearing emergency rescue clothing in hot environments, which could enhance occupational safety and health. Nonetheless, the study is not without its limitations. Firstly, the participants recruited were university students rather than emergency rescuers, the conclusions from this study may differ from the rescuers' due to individual differences such as age, body fitness, heat accumulation, and BMI. One notable limitation of this study is the relatively small and homogeneous sample size, consisting of 13 healthy male participants. While this sample size is consistent with similar pilot studies in thermal-physiological research, it limits the generalizability of the findings across populations with diverse physiological characteristics, such as females, older adults, or individuals with different fitness levels. Given these constraints, the results of this study should be interpreted as exploratory and hypothesis-generating. Future studies with larger, more diverse cohorts and power-informed sampling strategies will be essential to validate and expand upon these findings.

## 5 Conclusions

Human trials were performed in a climate chamber to investigate the ERC on physiological and perceptual responses in hot-humidity environments, the findings were summarized as follows:

- Compared with the CON group, ERC increased physiological responses in core temperature and mean skin temperature, but there was no significant trend in subjective perceptual responses, only differences were observed at specific time points.
- For the ERC trials, a positive correlation between PeSI and PSI and between mean skin temperature and thermal sensation vote was observed with *R* values of 0.93 and 0.94, respectively.
- PeSI has the potential to predict physiological burdens in hot environments, thereby offering a simple but effective approach to evaluating PSI and developing an early warning tool to reduce heat-related occupational health.

## Data Availability

The raw data supporting the conclusions of this article will be made available by the authors, without undue reservation.

## References

[1] UNDRR. The Human Cost of Disasters: An Overview of the Last 20 Years (2000-2019). (2020). Available online at: https://www.undrr.org/publication/human-cost-disasters-overview-last-20-years-2000-2019 (Accessed October 30, 2024).

[2] Zhang H Zheng Q Ke Y Wang H Wang M Ye Y. Design and performance evaluation of protective clothing for emergency rescue. Autex Res J. (2022) 22:1–10. 10.2478/aut-2020-0056

[3] Kim JH Coca A Williams WJ Roberge RJ. Subjective perceptions and ergonomics evaluation of a liquid cooled garment worn under protective ensemble during an intermittent treadmill exercise. Ergonomics. (2011) 54:626–35. 10.1080/00140139.2011.583362 21770750

[4] Hu Q Shen X Qian X Huang G Yuan M. The personal protective equipment (PPE) based on individual combat: a systematic review and trend analysis. Def Technol. (2023) 28:195–221. 10.1016/j.dt.2022.12.007

[5] Liu J Varghese BM Hansen A Zhang Y Driscoll T Morgan G. Heat exposure and cardiovascular health outcomes: a systematic review and meta-analysis. Lancet Planet Health. (2022) 6:E484–95. 10.1016/S2542-5196(22)00117-6 35709806

[6] ISO. ISO 7243:2017. Available online at: https://www.iso.org/standard/67188.html (Accessed February 13, 2025).

[7] Psychological impacts of disaster on rescue workers: a review of the literature. Int J Disaster Risk Reduct. (2018) 27:602–17. 10.1016/j.ijdrr.2017.10.020

[8] Pamuk G Cureklibatir Encan B Yildiz E. Thermal characteristics, mechanical and comfort properties of heat-protective textiles. Fibers Polym. (2023) 24:4457–68. 10.1007/s12221-023-00398-z

[9] Yu Z Liu J Suryawanshi A He H Wang Y Zhao Y. Thermal insulating and fire-retarding behavior of treated cotton fabrics with a novel high water-retaining hydrogel used in thermal protective clothing. Cellulose. (2021) 28:2581–97. 10.1007/s10570-020-03622-6

[10] Zhang Q Ma L Xue T Tian J Fan W Liu T. Flame-retardant and thermal-protective polyimide-hydroxyapatite aerogel fiber-based composite textile for firefighting clothing. Compos Part B Eng. (2023) 248:110377. 10.1016/j.compositesb.2022.110377

[11] Kim H. Manufacturing and properties of various ceramic embedded composite fabrics for protective clothing in gas and oil industries part I: anti-static and UV protection with thermal radiation. Coatings. (2023) 13:1481. 10.3390/coatings13091481

[12] Sukumar N Gananvel P Dharmalingam R Aruna S. Development of chemical protective clothing using multilayer fabric for hazardous chemicals handling. J Nat Fibers. (2022) 19:1265–80. 10.1080/15440478.2020.1764450

[13] Jussila K Valkama A Remes J Anttonen H Peitso A. The effect of cold protective clothing on comfort and perception of performance. Int J Occup Saf Ergon. (2010) 16:185–97. 10.1080/10803548.2010.11076838 20540839

[14] Teyeme Y Malengier B Tesfaye T Ciesielska I Musa A Van Langenhove L. A review of contemporary techniques for measuring ergonomic wear comfort of protective and sport clothing. Autex Res J. (2021) 21:377–93. 10.2478/aut-2019-0076

[15] Rouhani ST Fashandi H. Breathable dual-layer textile composed of cellulose dense membrane and plasma-treated fabric with enhanced comfort. Cellulose. (2018) 25:5427–42. 10.1007/s10570-018-1950-9

[16] Yang J Zhang Y Huang Y Chen W. Effects of liquid cooling garment on physiological and psychological strain of firefighter in hot and warm environments. J Therm Biol. (2023) 112:103487. 10.1016/j.jtherbio.2023.103487 36796928

[17] Richardson JE Capra MF. Physiological responses of firefighters wearing level 3 chemical protective suits while working in controlled hot environments. J Occup Environ Med. (2001) 43:1064–72. 10.1097/00043764-200112000-00008 11765677

[18] Nayak R Kanesalingam S Houshyar S Wang L Padhye R Vijayan A. Evaluation of thermal, moisture management and sensorial comfort properties of superabsorbent polyacrylate fabrics for the next-to-skin layer in firefighters' protective clothing. Text Res J. (2018) 88:1077–88. 10.1177/0040517517697640

[19] Fine B Kobrick J. Effect of heat and chemical protective clothing on cognitive performance. Aviat Space Environ Med. (1987) 58:149–54. 3827791

[20] Sawka MN Leon LR Montain SJ Sonna LA. Integrated physiological mechanisms of exercise performance, adaptation, and maladaptation to heat stress. Compr Physiol. (2011) 1:1883–928. 10.1002/j.2040-4603.2011.tb00385.x 23733692

[21] Tessier D. 4 - Testing thermal properties of textiles. In:Dolez P Vermeersch O Izquierdo V, editors. Advanced Characterization and Testing of Textiles. Cambridge: Woodhead Publishing (2018). p. 71–92. 10.1016/B978-0-08-100453-1.00005-2

[22] Havenith G Holmér I den Hartog EA Parsons KC. Clothing evaporative heat resistance–proposal for improved representation in standards and models. Ann Occup Hyg. (1999) 43:339–46. 10.1016/S0003-4878(99)00052-6 10481633

[23] Climate chamber study on thermal comfort of walking passengers at different moving speeds. Build Environ. (2022) 224:109540. 10.1016/j.buildenv.2022.109540

[24] Liu Z Yang H Wei X. Spatiotemporal variation in relative humidity in Guangdong, China, from 1959 to 2017. Water. (2020) 12:3576. 10.3390/w12123576

[25] Chan APC Yang Y. Practical on-site measurement of heat strain with the use of a perceptual strain index. Int Arch Occup Environ Health. (2016) 89:299–306. 10.1007/s00420-015-1073-7 26139094

[26] Selkirk GA McLellan TM Wong J. Active versus passive cooling during work in warm environments while wearing firefighting protective clothing. J Occup Environ Hyg. (2004) 1:521–31. 10.1080/15459620490475216 15238305

[27] Ryan GA Bishop SH Herron RL Katica CP Elbon BL Bosak AM. Ambient air cooling for concealed soft body armor in a hot environment. J Occup Environ Hyg. (2014) 11:93–100. 10.1080/15459624.2013.843782 24369931

[28] Ramanathan NL. A new weighting system for mean surface temperature of the human body. J Appl Physiol. (1964) 19:531–3. 10.1152/jappl.1964.19.3.531 14173555

[29] Moran DS Shitzer A Pandolf KB. A physiological strain index to evaluate heat stress. Am J Physiol. (1998) 275:R129–34. 10.1152/ajpregu.1998.275.1.R129 9688970

[30] Zhang H Arens E Huizenga C Han T. Thermal sensation and comfort models for non-uniform and transient environments: part I: local sensation of individual body parts. Build Environ. (2010) 45:380–8. 10.1016/j.buildenv.2009.06.018

[31] Borg GA. Psychophysical bases of perceived exertion. Med Sci Sports Exerc. (1982) 14:377–81. 10.1249/00005768-198205000-00012 7154893

[32] Tikuisis P McLellan TM Selkirk G. Perceptual versus physiological heat strain during exercise-heat stress. Med Sci Sports Exerc. (2002) 34:1454–61. 10.1097/00005768-200209000-00009 12218738

[33] American American Journal of Physiology-Regulatory Integrative and Comparative Physiology. A Physiological Strain Index to Evaluate Heat Stress. American Physiological Society. Available online at: https://journals.physiology.org/doi/full/10.1152/ajpregu.1998.275.1.R129 (Accessed July 8, 2025).

[34] Barnekow-Bergkvist M Aasa U Angquist KA Johansson H. Prediction of development of fatigue during a simulated ambulance work task from physical performance tests. Ergonomics. (2004) 47:1238–50. 10.1080/00140130410001714751 15370859

[35] Olesen BW Brager GS. A Better Way to Predict Comfort: The New ASHRAE Standard 55-2004. (2004). Available online at: https://escholarship.org/uc/item/2m34683k (Accessed July 8, 2025).

[36] Tang Y He Y Shao H Ji C. Assessment of comfortable clothing thermal resistance using a multi-scale human thermoregulatory model. Int J Heat Mass Transf. (2016) 98:568–83. 10.1016/j.ijheatmasstransfer.2016.03.030

[37] Lai D Zhou X Chen Q. Measurements and predictions of the skin temperature of human subjects on outdoor environment. Energy Build. (2017) 151:476–86. 10.1016/j.enbuild.2017.07.009

[38] Liu W Lian Z Deng Q Liu Y. Evaluation of calculation methods of mean skin temperature for use in thermal comfort study. Build Environ. (2011) 46:478–88. 10.1016/j.buildenv.2010.08.011

[39] Onofrei E Rocha AM Catarino A. Investigating the effect of moisture on the thermal comfort properties of functional elastic fabrics. J Ind Text. (2012) 42:34–51. 10.1177/1528083711425840

[40] Du C Li B Yu W Liu H Li C Yao R. Moisture in clothing and its transient influence on human thermal responses through clothing microenvironment in cold environments in winter. Build Environ. (2019) 150:1–12. 10.1016/j.buildenv.2018.12.066

[41] Kalazic A Brnada S Kis A. Thermal protective properties and breathability of multilayer protective woven fabrics for wildland firefighting. Polymers. (2022) 14:2967. 10.3390/polym14142967 35890743 PMC9317430

[42] Rowell L Brengelmann G Blackmon J Murray J. Redistribution of blood flow during sustained high skin temperature in resting man. J Appl Physiol. (1970) 28:415. 10.1152/jappl.1970.28.4.415 5437428

[43] Bridge CA Jones MA Drust B. Physiological responses and perceived exertion during international Taekwondo competition. Int J Sports Physiol Perform. (2009) 4:485–93. 10.1123/ijspp.4.4.485 20029099

[44] Larsen B Netto K Skovli D Vincs K Vu S Aisbett B. Body armor, performance, and physiology during repeated high-intensity work tasks. Mil Med. (2012) 177:1308–15. 10.7205/MILMED-D-11-00435 23198506

[45] Bach AJE Maley MJ Minett GM Zietek SA Stewart KL. Stewart IB. An evaluation of personal cooling systems for reducing thermal strain whilst working in chemical/biological protective clothing. Front Physiol. (2019) 10:424. 10.3389/fphys.2019.00424 31031643 PMC6474400

[46] Carballo-Leyenda B Villa JG López-Satué J Collado PS Rodríguez-Marroyo JA. Fractional contribution of wildland firefighters' personal protective equipment on physiological strain. Front Physiol. (2018) 9:1139. 10.3389/fphys.2018.01139 30154736 PMC6103002

[47] Simpson KM Munro BJ Steele JR. Does load position affect gait and subjective responses of females during load carriage? Appl Ergon. (2012) 43:479–85. 10.1016/j.apergo.2011.07.005 21831354

[48] Savage RJ Lord C Larsen BL Knight TL Langridge PD Aisbett B. Firefighter feedback during active cooling: a useful tool for heat stress management? J Therm Biol. (2014) 46:65–71. 10.1016/j.jtherbio.2014.10.003 25455942

